# Small Animal Model of Post-chemotherapy Tuberculosis Relapse in the Setting of HIV Co-infection

**DOI:** 10.3389/fcimb.2020.00150

**Published:** 2020-04-16

**Authors:** Matthew B. Huante, Tais B. Saito, Rebecca J. Nusbaum, Kubra F. Naqvi, Sadhana Chauhan, Robert L. Hunter, Jeffrey K. Actor, Jai S. Rudra, Mark A. Endsley, Joshua G. Lisinicchia, Benjamin B. Gelman, Janice J. Endsley

**Affiliations:** ^1^Department of Microbiology and Immunology, University of Texas Medical Branch, Galveston, TX, United States; ^2^Department of Pathology, University of Texas Medical Branch, Galveston, TX, United States; ^3^Department of Microbiology, University of Pennsylvania, Philadelphia, PA, United States; ^4^Department of Pathology and Laboratory Medicine, University of Texas Health Sciences Center, Houston, TX, United States; ^5^Department of Biomedical Engineering, Washington University in St. Louis, St. Louis, MO, United States

**Keywords:** TB, HIV, TB and HIV co-infection, TB relapse, pathology, TB chemotherapy, immune response, tuberculosis

## Abstract

Tuberculosis relapse following drug treatment of active disease is an important global public health problem due to the poorer clinical outcomes and increased risk of drug resistance development. Concurrent infection with HIV, including in those receiving anti-retroviral therapy (ART), is an important risk factor for relapse and expansion of drug resistant *Mycobacterium tuberculosis* (*Mtb*) isolates. A greater understanding of the HIV-associated factors driving TB relapse is important for development of interventions that support immune containment and complement drug therapy. We employed the humanized mouse to develop a new model of post-chemotherapy TB relapse in the setting of HIV infection. Paucibacillary TB infection was observed following treatment with Rifampin and Isoniazid and subsequent infection with HIV-1 was associated with increased *Mtb* burden in the post-drug phase. Organized granulomas were observed during development of acute TB and appeared to resolve following TB drug therapy. At relapse, granulomatous pathology in the lung was infrequent and mycobacteria were most often observed in the interstitium and at sites of diffuse inflammation. Compared to animals with HIV mono-infection, higher viral replication was observed in the lung and liver, but not in the periphery, of animals with post-drug TB relapse. The results demonstrate a potential role for the humanized mouse as an experimental model of TB relapse in the setting of HIV. Long term, the model could facilitate discovery of disease mechanisms and development of clinical interventions.

## Introduction

Tuberculosis (TB) caused by *Mycobacterium tuberculosis* (*Mtb*) infection is the leading cause of infectious disease-related mortality, with the World Health Organization estimating 10 million cases of TB and 1.45 million deaths in 2018 (WHO, 2019). Completion of combination drug therapy leads to clinical cure in most subjects, however, a spectrum of incomplete cures that range from latent infection to active TB occur frequently (Dooley et al., 2011; Rockwood et al., 2016; McIvor et al., 2017) and play an important role in the global TB burden (Chao and Rubin, 2010; Mirsaeidi and Sadikot, 2018). In those with bacteriological evidence of cure following drug therapy, recurrent TB can occur due to endogenous reactivation (relapse) or re-infection. DNA fingerprint analysis of case samples demonstrates that at least half of recurrent TB in endemic sites occurs due to relapse of a persisting *Mtb* strain and not a new infection (Marx et al., 2014). Recurrent TB that develops within the first year following the completion of antibiotic treatment is most often a relapse event (Sonnenberg et al., 2001; Shen et al., 2006; Crampin et al., 2010; Narayanan et al., 2010; Unis et al., 2014) and is associated with greatly increased risk for resistance to front line TB drugs (Yoshiyama et al., 2004; Munje et al., 2015). The emergence of multi (MDR)- and extensively (XDR)-drug resistant isolates in a growing number of geographical regions is an important factor contributing to the global health crisis of TB (WHO, 2019).

Risk factors associated with TB recurrence include cavitary disease, sputum positivity after the intensive phase of treatment (Lee and Kim, 2014), HIV infection, malnutrition, or treatment with immunosuppressive therapies (see reviews; Braun et al., 1989; Cisneros and Murray, 1996; Douglas et al., 1996; Rajagopalan and Yoshikawa, 2000; Bresnihan and Cunnane, 2003; Gideon and Flynn, 2011; Ernst, 2012; Esmail and Barry, 2012). Among these risk factors, HIV infection is one of the most significant due to the epidemiological overlap of the afflicted populations, loss or dysfunction of immunity, and several other poorly understood mechanisms for synergistic pathogenesis (Pulido et al., 1997; Pawlowski et al., 2012; Unis et al., 2014). People living with HIV (PLWH) are more likely to experience: TB chemotherapy failure, TB relapse following treatment, and reactivation of latent TB (Khan et al., 2010; Unis et al., 2014; Trinh et al., 2015).

Clinical management of TB and HIV co-infection is challenging and can impact TB treatment outcomes (Mirsaeidi and Sadikot, 2018; Tornheim and Dooley, 2018). There are several clinically relevant scenarios whereby co-infection with HIV could increase risk for TB relapse including: non-diagnosed HIV infection, new HIV infection during or following the standard 6 month TB treatment phase, or poor ART access or compliance during or following TB chemotherapy. In endemic regions, the potential for HIV-associated factors to impact treatment is even greater for those undergoing drug therapy for multi-drug resistant TB due the 18–24 month treatment period (Falzon et al., 2017). The strong association between TB and HIV includes biological mechanisms of pathogen synergy as well as many non-biological factors (e.g., socioeconomic) and clinical issues as recently reviewed (Huante et al., 2019). A greater understanding of the complex interplay between *Mtb* and HIV in the setting of *in vivo* drug treatment is needed to inform development of new approaches to reduce TB relapse.

An animal model of post-drug TB relapse in the setting of HIV, or SIV, has not been described to date. As a result, our current understanding is based on extrapolations from clinical outcomes in *Mtb*/HIV co-infected human subjects and models of latent TB infection (LTBI) reactivation due to SIV infection in non-human primates (NHP) (Diedrich et al., 2010; Mehra et al., 2011). The loss of CD4^+^T cells is a well-established deficiency in PLWH whose HIV infection has progressed to the acquired immune deficiency syndrome (AIDS) stage. Reduced production of effector molecules (e.g., IFN-γ, TNF-α) due to the effects of HIV on both loss and cellular function of CD4^+^T cells has been linked to poor immune containment of *Mtb* infection (see reviews Diedrich and Flynn, 2011; Pawlowski et al., 2012). Increasingly, though, the risk for TB relapse has been shown to occur along a spectrum of CD4^+^T cell loss, including in those subjects virally suppressed following ART (Sonnenberg et al., 2005; Walker et al., 2013). As observed in human subjects, reactivation of LTBI due to SIV in NHP has been shown to be associated with both CD4^+^T cell loss-dependent and independent mechanisms (Diedrich et al., 2010; Foreman et al., 2016). Development of a small animal model amenable to infection with human HIV isolates would facilitate mechanistic studies to understand the immunological and microbiological alterations that promote post-drug TB relapse and provide a system to test clinical interventions.

Our results demonstrate the potential to use the human immune system (HIS) mouse model of TB (Calderon et al., 2013; Nusbaum et al., 2016) to investigate TB relapse *in vivo* in animals co-infected with HIV-1. Consistent with observations of greater TB relapse in PLWH, infection of HIS mice with HIV increased *Mtb* burden following drug treatment with RIF and INH. The increased HIV viral load observed in animals with TB relapse also further suggests that *Mtb* infection may provide a favorable niche for viral replication in tissue compartments. These results support further exploration of the HIS mouse models for discovery of disease mechanisms for TB relapse due to HIV co-infection and as a platform for development and testing of therapeutic interventions. The current model investigated the effects of HIV infection when introduced at the end of TB drug therapy. Further adaptation could facilitate mechanistic investigations of several important clinical scenarios including the effects of chronic HIV, or HIV infection suppressed by ART, on TB treatment outcomes.

## Materials and Methods

### Ethics Statement

The experimental procedures using mice were approved by the University of Texas Medical Branch Institutional Animal Care and Use Committee under protocols 1604017 and 1501001. Mice were housed in HEPA filtered cages in a climate controlled facility designed to maintain optimal temperature, humidity, and light cycle. Food and water were provided *ad libitum*, in addition to nesting material. Animals were monitored daily by trained animal resource center staff with oversight by staff veterinarians. Upon showing signs of severe disease beyond the point of recovery the mice were humanely euthanized. De-identified human tissue specimens used in the development of humanized mice were obtained from Advanced Bioscience Resources, Alameda, CA as previously described (Calderon et al., 2013). Tissues were confirmed to be free of specific infectious agents including HIV, Hepatitis B, and Hepatitis C virus.

### Generation of Humanized Mice

All infection experiments were performed using HIS bone marrow, liver, and thymus (or BLT) mice that were generated using the NOD-SCID/$\gamma_{\text{c}}^{\text{null}}$ (NSG) mouse as previously described (Calderon et al., 2013; Nusbaum et al., 2016). Reconstitution of a human immune system was validated using flow cytometric detection of human leukocytes including T cell (CD4 and CD8) and myeloid cell (monocytes, macrophages, and dendritic cell) populations as we described (Calderon et al., 2013). Animals that displayed evidence of graft vs. host disease (GVHD) such as allopecia that can occur in extended studies with HIS mouse models, as described (Greenblatt et al., 2012; Karpel et al., 2015), were excluded from analysis. Losses/exclusions due to GVHD were 30–40% of animals in the two studies due to the length of the studies. Mice from two HIS mouse groups were used to establish paucibacillary TB and assess the potential to study relapse following HIV infection. Excluding animals that developed GVHD, data from 12 animals is shown in study 1 including non-infected control (*n* = 2), post-infection and post-rifampicin confirmation of infection and bacterial reduction (*n* = 4, with 2 animals per timepoint), TB relapse (*n* = 3) and TB/HIV relapse (*n* = 3) groups. In study 2, excluding GVHD animals, data analysis is from 18 animals including non-infected control (*n* = 3), post-infection and post-rifampicin confirmation of infection and bacterial reduction (*n* = 4, with 1 animal per timepoint), TB relapse (*n* = 4), TB/HIV relapse (*n* = 4), and HIV infection (*n* = 3) groups.

### Infections With *Mtb*

Mycobacterial infections were performed after assignment of HIS mice to treatment groups including mock (PBS) or *Mtb* infection using the H37Rv strain via an intranasal (i.n.) route of infection as described (Calderon et al., 2013). A growth stock of *Mtb* H37Rv was propagated in Middlebrook 7H9 and suspended in PBS for use as inoculum to deliver *Mtb* to the lung compartment by intranasal distillation of 40 μl (20 μl/nare). Bacterial enumeration was performed using limiting dilutions of the innoculum plated on 7H11 agar plates to estimate the *Mtb* infection dose that was administered as described (Endsley et al., 2006). The estimated i.n. dose, based on CFU of plated inoculum was 10^2^ CFU/mouse in study 1, and 10^3^ CFU/mouse in study 2. Tissues from non-infected control mice generated from the same HIS production groups were used to establish normal tissue histology and immune analyte baselines. The HIS mice infected with only HIV-1 in study 2 received mock *Mtb* infection (PBS).

### Drug Treatment to Generate Paucibacillary Infection

A Cornell-like model of paucibacillary TB that was previously described in the C57BL/6J mouse (Radaeva et al., 2005) was reproduced in HIS mice. Following development of clinical signs of active TB at 8 and 4 weeks post-infection in study 1 and 2, respectively, TB chemotherapy was initiated for 8 weeks. Treatment consisted of daily oral gavage for seven days a week with 750 ug of both rifampin (RIF) and isoniazid (INH) purchased from Sigma-Aldrich and dissolved in 100 μl of sterile water. Stock solutions of RIF were dissolved in a low volume DMSO and final concentration of DMSO in the delivered dose was <0.01%. Chemotherapy was provided to all treatment groups (including non-infected and HIV-infected groups) to control for drug and carrier effects and potential carryover of weak antiviral activity that is described for RIF (Clark, 1971; Moshkowitz et al., 1971). Upon development of clinical signs (e.g., weight loss) following 4–8 weeks of *Mtb* infection and upon completion of 8 weeks of TB chemotherapy, the bacterial burden was determined in randomly selected animals using CFU enumeration of disrupted tissue. Tissue specimens were also preserved for histopathology and histochemistry analysis.

### Infections With HIV

At the completion of TB chemotherapy, HIS mice were infected with 2,500 TCID_50_ of HIV-1 (JR-CSF strain, UCLA Center for AIDS Research) or mock infected (PBS) as we previously described (Nusbaum et al., 2016). Virus stock was diluted in PBS and infections performed by i.v. delivery of 100 μl via the tail vein. Blood was collected prior to infection and at necropsy and plasma was frozen for subsequent assessment of viral infection.

### Determination of Viral and Bacterial Burden

Animals were humanely euthanized at the designated timepoints or the observations of clinical signs of disease (e.g., weight loss, lethargy). Randomly selected animals were euthanized prior to initiation of TB drug therapy to confirm development of active *Mtb* infection and following 8 week of drug therapy to demonstrate drug efficacy. The left lung lobe and the median liver lobe was preserved in formalin and embedded in paraffin for use in hisopathological analysis and detection of acid fast bacilli (AFB) using the Ziehl-Neelson method. The right lung superior, inferior, and post-caval lung lobes and the left liver lobe were disrupted using a tissue grinder (Kendall, Inc) to determine mycobacterial burden by CFU enumeration following limiting dilution and growth on 7H11 agar plates as described (Endsley et al., 2006). At necropsy, blood and tissues (lung and liver) were collected from the mice. Tissue were disrupted in 1 ml of sterile PBS using a tissue grinder (Kendell, Inc) and aliquots of the tissue homogenate were used for limiting dilution analysis of CFU as described (Endsley et al., 2006). The limit of detection of *Mtb* CFU using this enumeration method is 30 bacilli. Supernatants collected from tissue homogenates in study 2 were used for assessment of viral load and cytokine and chemokine profiles. Viral load was determined by measurement of HIV p24 capsid protein levels in the plasma, lung, and liver, using an ELISA (Zeptometrix Corporation).

### Histopathology and RNA Scope Analysis

Formalin-fixed and paraffin embedded tissue sections were cut, dewaxed, and stained using hematoxylin and eosin (H&E) for histopathology analysis (UTMB Research Histopathology Core Facility) and the Ziehl-Neelson method to detect AFB. Additional tissue sections were used for analysis of viral RNA in the lung using RNA scope (ACD Bio) using *in situ* hybridization with probes specific to the HIV-1 *gag* gene in accordance with manufacturer's instructions.

### Cytokine and Chemokine Quantification

Supernatants harvested from disrupted lung were stored at −80°C and inactivated by exposure to 5 MRAD γ-irradiation on dry ice using a JL Shepherd Model 109–68 Cobalt-60 Research Irradiator (JL Shepherd & Associates, San Fernando, CA). Sterility was confirmed by lack of CFU following 3 weeks of growth on 7H11 agar. Analysis of lung cytokines and chemokines affected by treatment was performed with a human multiplex ELISA (Bio-rad Bio-plex Pro™ human cytokine 27-plex kit) according to the manufacturer's instructions as previously described (Nusbaum et al., 2016). Results from molecules with cross-reactivity between human detection reagents and mouse cytokines (e.g., VEGF, IL-13) were excluded from the analysis to reduce confounding outcomes. Values that were out of range high were not observed in any of the samples tested. The out of range low values were set to zero and data from tissue analytes where many values were out of range low are not presented. Analytes were quantified by generating a standard curve using validated standards and values were determined by linear regression to the standard curve as described (Nusbaum et al., 2016).

### Statistics

Microbiological and immunological data from animal studies are shown as mean ± SEM. One-way ANOVA followed by a Bonferroni's multiple comparison test was used for multiple group comparisons (GraphPad Software v7.0).

## Results

### Paucibacillary TB and Granuloma Resolution Following TB Chemotherapy

**Figure 1:**
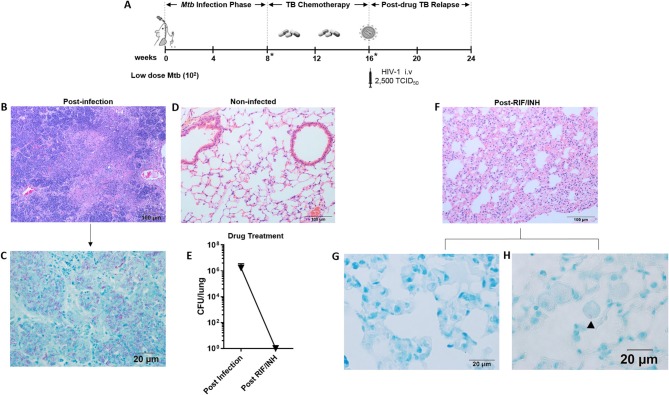
Paucibacillary TB and granuloma resolution following TB chemotherapy. **(A)** Experimental design for study 1. HIS BLT mice were generated and infected i.n. with 10^2^ CFU of *Mtb* H37Rv for 8 weeks followed by treatment with RIF and INH by oral gavage (750 μg/day) for 8 weeks. Drug treatment was terminated and HIS mice were assigned to i.v. infection with 2,500 TCID_50_ of HIV-1 (JR-CSF) or mock (PBS) using 3 mice per group. Relapse of TB was assessed at 8 weeks post-HIV infection. Infection with *Mtb* and drug efficacy was confirmed by CFU enumeration in tissues of 2 animals per designated (*) time point with a limit of detection of 30 organisms. Representative images from histological findings as visualized with H&E and Ziehl-Neelson staining and brightfield microscopy. **(B)** Granulomatous pathology characteristic of active TB in HIS mouse lung and **(C)** corresponding AFB present in the inflamed tissue. **(D)** Healthy lung from a non-infected mouse. **(E)** Mycobacterial burden in the lung, as determined by CFU enumeration following 8 weeks of infection and 8 weeks of drug treatment with RIF and INH. **(F)** Residual interstitial inflammation observed in lung following drug treatment, including **(G)** AFB negative inflammatory cells frequently observed in interstitium, and **(H)** Rare AFB observed in alveolar macrophage.

**Figure 2:**
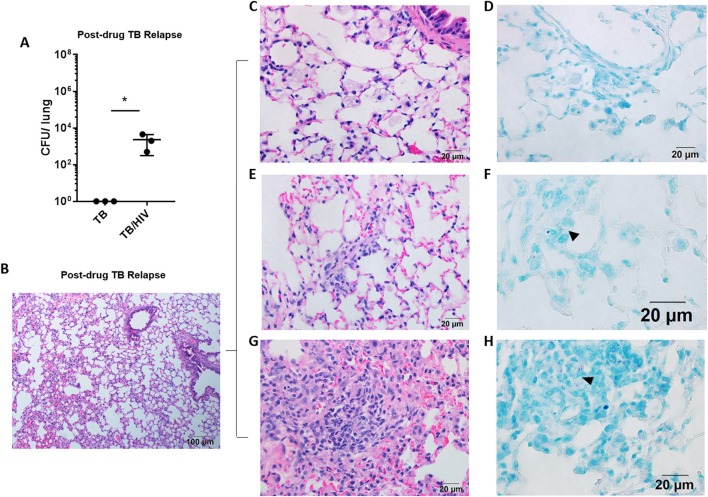
Pulmonary TB relapse in HIS mice co-infected with HIV-1. Pulmonary TB relapse outcomes in the setting of HIV infection in study 1. Following establishment of paucibacillary *Mtb* infection with chemotherapy, HIS mice were co-infected i.v. with mock (PBS) or HIV-1 (2,500 TCID_50_ JR-CSF). Experiments were terminated at 8 weeks post HIV or mock infection to assess TB relapse. **(A)** Lung bacterial burden during the relapse phase in TB and TB/HIV infection groups. Histological appearance of lung as shown with representative images from animals in both TB and TB/HIV groups. **(B)** Non-remarkable lung with occasional pockets of inflammation and frequent areas of interstitial inflammation that include features illustrated in C-H. **(C)** Foamy alveolar macrophages that are **(D)** lacking AFB. **(E)** Paraseptal and interstitial inflammation with **(F)** occasional AFB positive (arrowhead) inflammatory cells in interstitium. **(G)** Larger areas of inflammation containing **(H)** pockets of AFB (arrowhead). Data shown in **(A)** is the mean ± SEM and statistically significant differences between treatment groups are shown as *p < 0.05.

We previously developed a HIS mouse model of TB (Calderon et al., 2013) and acute TB/HIV co-infection (Nusbaum et al., 2016) in the BLT mouse. Here we further adapted this *in vivo* system to model TB relapse in a Cornell-like model of disease (Radaeva et al., 2005) in order to permit exploration of the effects of HIV co-infection on relapse. The experimental approach for outcomes following low dose infection (study 1) is diagrammed in Figure 1A. The data shown in Figures 1, 2, and Supplemental Figure 1 were from experiments conducted with this design.

Representative images of the pathology observed among the mice following low dose *Mtb* infection and treatment with TB chemotherapy is shown in Figure 1. Following 8 weeks of *Mtb* infection, the lungs of HIS mice were characterized by large areas of granulomatous inflammation including some areas of necrosis and caseous necrosis (Figure 1B). Within the granulomatous lesions, bacilli were abundant as detected with Ziehl-Neelson staining for AFB (Figure 1C). As shown in Figure 1D, normal lung architecture is observed in non-infected HIS mice. Assessment of mouse lung tissue by CFU enumeration (Figure 1E) confirmed the presence of an established infection at 8 weeks post-*Mtb* infection. The bacterial load in the mice was below the limit of detection, however, following 8 weeks of daily oral TB chemotherapy with 750 μg daily RIF and INH (Figure 1E).

An interesting observation made in the lungs of HIS mice 8 weeks after TB chemotherapy was the lack of residual granulomatous lesions. Areas of persisting interstitial inflammation as shown in Figure 1F, however, were frequently observed, and were generally devoid of detectable AFB as shown in Figure 1G. Very rare bacilli were observed in brightfield microscopy analysis of lung following TB chemotherapy, as illustrated with one AFB detected in a foamy alveolar macrophage in Figure 1H. Taken together, these results indicate that a paucibacillary state of *Mtb* infection in HIS mice occurs coincident with apparent resolution of granulomatous lung pathology following drug treatment.

### Pulmonary TB Relapse in HIS Mice Co-infected With HIV-1

Following 8 weeks of TB drug therapy, HIS mice were subsequently assigned to TB and TB/HIV treatment groups as diagrammed in Figure 1A. The animals in the TB/HIV group were infected i.v. with 2,500 TCID_50_ of a human clinical HIV-1 isolate (JR-CSF) as diagrammed in Figure 1. At 8 weeks following cessation of TB drug therapy and infection with HIV, the development of relapse was assessed. As shown in Figure 2A, CFU enumeration revealed a significant increase in pulmonary mycobacterial burden of mice with HIV, as compared to mock, co-infection. In lungs of HIV naïve mice, bacilli were below the limit of detection for growth on solid media.

Analysis of the lung histology demonstrated that the interstitial inflammation observed at the completion of drug treatment (Figure 1F) may have partially resolved by 8 weeks after drug treatment cessation (Figure 2B). Surprisingly, a fairly similar histological appearance was observed in the lung of mice from both the TB and TB/HIV treatment groups as shown with representative images in Figures 2B–H. This included areas of normal alveolar architecture and areas with small foci of inflammation in the alveolar walls as shown in Figure 2B. Foamy alveolar macrophages (Figure 2C) that were lacking AFB (Figure 2D) were also observed. Areas with interstitial or paraseptal inflammatory cells (Figure 2E) were also frequently seen. Rare AFB were observed at these sites of mild inflammatory cell accumulation in the lung of mice from both the TB and TB/HIV groups as shown in Figure 2F. Some areas of moderate inflammation associated with small pockets of AFB were occasionally observed as shown in Figures 2G,H.

### Hepatic TB in HIS Mice Following Drug Treatment and HIV Infection

To determine whether HIV infection may promote TB growth or regrowth at a common site of *Mtb* dissemination and viral replication in the HIS mouse, an analysis of hepatic tissue was performed. HIV infects liver tissue (Housset et al., 1990) and extra-pulmonary TB presentation is more commonly observed in PLWH as compared to those with negative HIV status (Leeds et al., 2012). Following low dose *Mtb* infection in study 1, dissemination of *Mtb* to the liver was observed (Supplemental Figure 1A) at 8 weeks post-infection. Consistent with this outcome, small AFB positive lesions were a frequent observation in microscopy analysis of liver specimens as shown in Supplemental Figures 1B,C.

Similar to the lung, chemotherapy with RIF and INH reduced culturable bacilli in the liver to below the limit of detection by 8 weeks (Supplemental Figure 1A). The liver was mostly non-remarkable following drug treatment (Supplemental Figure 1D) and AFB were not observed in liver parenchyma (Supplemental Figure 1E). In contrast to the lung, culturable bacilli were detected in the liver of animals from both the TB and TB/HIV groups at 8 weeks post-drug cessation (Supplemental Figure 1F) although no significant treatment differences were observed. The liver tissue from both treatment groups was generally unremarkable at this endpoint (Supplemental Figure 1G) and only rare AFB were detected (Supplemental Figure 1H). These data suggest the HIS mouse can effectively model the effects of HIV infection in the liver on TB relapse at sites of dissemination.

### Pulmonary TB Relapse Following High Dose *Mtb* Infection

**Figure 3:**
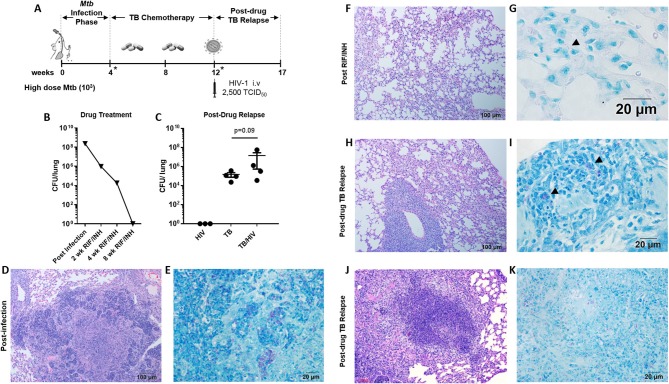
Pulmonary TB relapse following high dose *Mtb* exposure and HIV infection **(A)** Experimental overview for study 2. HIS BLT mice were infected i.n. with an estimated 10^3^ CFU of *Mtb* H37Rv for 4 weeks and subsequently treated with RIF and INH by oral gavage (750 μg/day) for 8 weeks. Drug treatment was terminated and HIS mice were assigned to i.v. infection with 2,500 TCID_50_ of HIV-1 (JR-CSF) or mock (PBS) using 4 HIS mice per group. Relapse of TB was assessed at 5 weeks post-HIV infection due to observation of signs of disease (e.g., weight loss). *Denotes confirmation of infection and drug activity in selected animals. **(B)** Confirmation of infection and drug efficacy prior to, during, and at the completion of TB chemotherapy (*n* = 4, with 1 animal per time point). **(C)** Bacterial burden 5 weeks post-HIV or mock infection in the TB and TB/HIV groups. Histological appearance of tissue from HIS mice following *Mtb* infection, drug treatment with RIF/INH, and at TB relapse is shown in **(D–K)**. **(D)** Granulomatous inflammation at 4 weeks post-infection with *Mtb* and **(E)** abundant AFB positive areas within the granuloma. **(F)** Residual interstitial inflammation and **(G)** rare AFB (arrowhead) in inflammatory cells in the interstitium, after 8 weeks of RIF and INH. Similar histological appearance of lung following relapse in TB and TB/HIV experimental groups is shown with representative images in **(H–K)**. **(H)** Frequently observed areas that include non-remarkable lung, interstitial inflammation, and perivascular inflammation and **(I)** AFB in small pockets of inflammation. **(J)** Infrequent granulomatous lesion and **(K)** AFB in a granuloma center.

A subsequent experiment (study 2) was conducted as detailed in Figure 3A in which sufficient HIS mice were generated from the same tissue source to permit inclusion of an HIV mono-infection group. This study differs from study 1 in that animals were infected with a higher estimated dose (10^3^ CFU) of *Mtb*. The shorter treatment phases shown in Figure 3A are due to the associated acceleration of TB disease progression that required earlier drug intervention following *Mtb* infection, and earlier endpoint collection in the relapse phase, respectively. As shown in Figure 3B, treatment with RIF and INH progressively reduced the pulmonary bacterial load to below the limit of detection by 8 weeks. In contrast to study 1, relapse TB in study 2 was indicated by detection of culturable bacilli in both the TB and TB/HIV groups (Figure 3C). A trend toward increased pulmonary burden (*p* = 0.09) was observed in lung of mice from the TB/HIV group, although this effect did not reach significance.

Active TB, as characterized by significant areas of granulomatous inflammation and abundant AFB in lung tissue was observed 4 weeks post-infection (Figures 3D,E) and was similar to observations in study 1 (Figures 1B,C) at 8 weeks post-infection. At 4 weeks into the 8 week drug treatment, diffuse inflammation was the most common finding as shown in Supplemental Figure 2A while small clusters of AFB were visible throughout the inflamed areas (Supplemental Figure 2B). By 8 weeks of RIF and INH treatment (Figure 3F), a pattern of interstitial inflammation similar to study 1 (Figure 1F) was observed. Rare AFB were seen in cells within these pockets of inflammation (Figure 3G), consistent with the observations in study 1 (Figure 2H).

At relapse, the histological appearance of the lung tissue in mice from the TB and TB/HIV groups was similar. Areas characterized as non-remarkable or as having mild inflammatory infiltrate were observed similar to study 1. In contrast to study 1, larger areas of interstitial and perivascular inflammation (Figure 3H) and infrequent areas of granulomatous inflammation (Figure 3J), were also noted. Small pockets of AFB were observed in areas of diffuse inflammation (Figure 3I) while more abundant AFB were seen in granulomas (Figure 3K).

### Hepatic Dissemination and Relapse Following High Dose *Mtb* Infection

**Figure 4:**
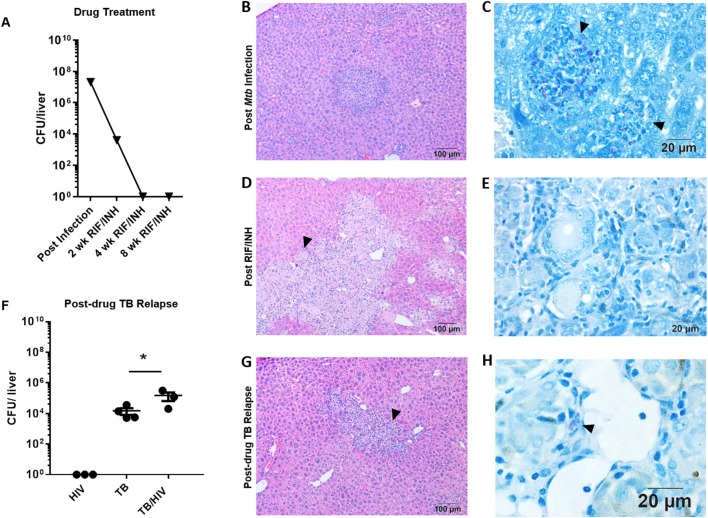
Hepatic TB relapse in the setting of HIV co-infection. Infection, drug treatment and TB relapse outcomes in liver of HIS mice given high dose (10^3^ CFU) *Mtb* infection in study 2. **(A)** CFU in the liver 4 weeks after i.n. infection with *Mtb* and after 2, 4, and 8 weeks of TB chemotherapy with RIF/INH. **(B)** Inflammatory foci containing **(C)** clusters of AFB (arrowheads) in the liver of mice following infection with *Mtb*. **(D)** Lesions with frequent multi-nucleated giant cells (arrowheads) observed following 8 weeks of TB chemotherapy which **(E)** lack detectable AFB. **(F)** Hepatic mycobacterial burden (CFU) in the TB relapse phase in HIV mono-infected, and *Mtb* and drug treated mice with mock (TB) or HIV (TB/HIV) co-infection. **(G)** Inflammatory foci similarly evident in liver from both TB and TB/HIV relapse in which **(H)** rare AFB are observed in the TB relapse phase. Data in B are means ± SEM with statistically significant differences between treatments shown as *p < 0.05.

Treatment with RIF and INH progressively reduced the mycobacterial burden in the liver to below the limit of detection by 8 weeks (Figure 4A). Organized inflammatory lesions were frequently observed in the liver parenchyma (Figure 4B) and contained AFB positive cells (Figure 4C). Following drug treatment, lesions containing inflammatory cells and multinucleated giant cells (MGC) were a common finding (Figure 4D). MGC are a characteristic feature of TB pathology that is not observed in several small animal models. Similar to study 1, AFB were not detected in these liver lesions (Figure 4E) or other areas of the liver at 8 weeks post-drug treatment.

Relapse was observed in liver of mice from both the TB and TB/HIV groups and a significant increase in CFU was observed due to HIV infection (Figure 4F). A caveat to note is that liver CFU data from one animal in the TB/HIV group was lost due to fungal contamination of the agar plate. The lung CFU result from this mouse was intermediate among the four animals in the TB/HIV group. In contrast to study 1 (Supplemental Figure 1), residual inflammation including the presence of MGC was observed in liver of animals from both groups (Figure 4G) post-drug therapy. MGC were also observed in the liver of a mouse harvested after 4 weeks of RIF and INH treatment as shown in Supplemental Figure 2C. At this stage of treatment, only rare AFB+ cells were observed in hepatic tissue (Supplemental Figure 2D). Despite the differences in liver bacterial burden in the relapse phase (Figure 4F), similar histological features were seen among tissue from animals in both the TB and TB/HIV treatment groups (Figure 4G) including the presence of MGC and cells containing AFB (Figure 4H).

### HIV Replication in Lung and Liver During TB Relapse

**Figure 5:**
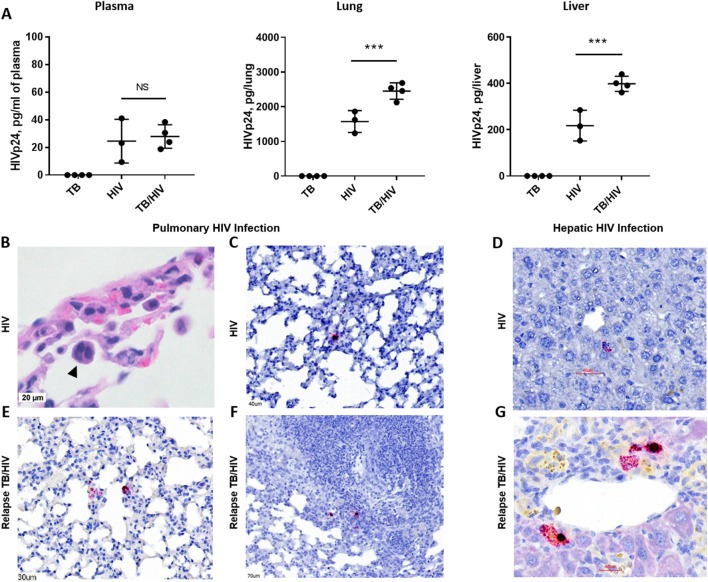
HIV replication in lung and liver during TB relapse. Viral load and distribution of HIV+ cells in the periphery and tissues in the TB relapse phase in study 2. **(A)** Viral load in the plasma, lung, and liver as measured by ELISA (Zeptometrix) specific to HIV p24 capsid protein. **(B)** Detection of a cellular syncytia (arrowhead) characteristic of HIV infection as observed in bright field microscopy following visualization with H&E. **(C–G)** HIV infected cells were detected with RNA-Scope specific to HIV gag. **(C,D)** HIV+ cells in the lung interstitium and near portal tracts in the liver of a HIS mouse with HIV mono-infection. **(E–G)** HIV+ cells were observed in inflamed interstitium **(E)** and in the periphery of TB lesions **(F)**, as well as near blood vessels in the liver **(G)**, of TB/HIV co-infected mice at TB relapse. Data shown in **(A)** are means ± SEM with statistically significant differences between indicated treatment groups shown as ***p < 0.001.

Increased viral replication has been described in the bronchoalveolar lavage of those with TB and HIV co-infection (Nakata et al., 1997). To determine if *Mtb* infection could alter viral replication in the periphery or tissues, the viral load and distribution of cells harboring viral RNA was assessed in HIS mice (Figure 5). An HIV mono-infection treatment group, naïve for *Mtb* exposure, was mock infected i.n. with PBS, treated with RIF/INH to control for potential drug effects, and subsequently infected with HIV. Similarly, control animals naïve for *Mtb* and HIV infection were also provided the RIF and INH regimen and tissues collected at the study end to provide baseline values. It is important to note that animals in the non-infected and HIV mono-infection treatment groups were generated in the same HIS mouse production batch (i.e., matched tissues/cells) as the other animals used in study 2.

As shown in Figure 5A, productive infection with HIV-1 was observed in blood and tissues of HIS mice from both the HIV and TB relapse/HIV groups at 5 weeks post HIV infection. Plasma levels of HIV proteins measured by a commercial diagnostic ELISA did not differ between HIV and TB/HIV infection groups (Figure 5A). Interestingly, the viral load was significantly increased in both the lung and the liver of mice with TB/HIV co-infection compared to the group mono-infected with HIV (Figure 5A).

Histopathology and RNA scope analysis were employed to further characterize HIV infection of the lung and liver. As shown in Figure 5B, cellular syncytia due to HIV infection was observed in bright field microscopy analysis of HIS mouse lung. This is an important cellular pathology observed in human tissue of those with HIV and is associated with cell to cell transfer of virus (Bracq et al., 2017) that we now demonstrate in the HIS mouse. RNA scope analysis further demonstrated limited areas of viral RNA in lung and liver of HIV mono-infected mice that were observed to be randomly distributed throughout the lung parenchyma (Figure 5C) and near portal tracts in the liver (Figure 5D). At TB relapse in co-infected animals, viral RNA was also detected in the lung parenchyma, and further found to be localized to interstitial spaces characterized by mild inflammatory infiltrate (Figure 5E), as well as the periphery of TB lesions (Figure 5F). In the liver of co-infected mice, HIV RNA was observed near portal tracts and in proximity to vessels (Figure 5G).

### Pulmonary Immune Microenvironment of TB and TB/HIV Relapse

**Figure 6:**
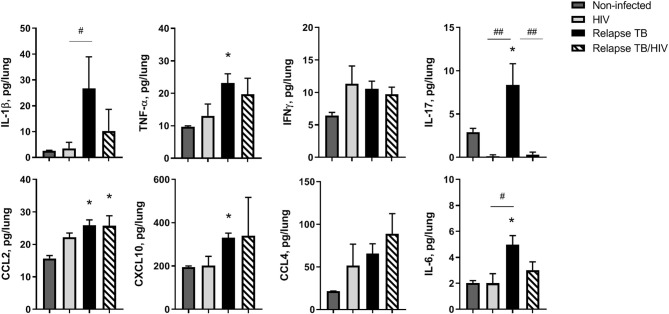
Pulmonary immune microenvironment of TB and TB/HIV relapse. Lung supernatants were harvested from disrupted lung tissue to assess differences in the immune microenvironment at TB relapse, in the presence or absence of HIV. Analysis of lung cytokines and chemokines was performed with a human multiplex ELISA (Bio-rad Bio-plex Pro™ human cytokine 27-plex kit. Shown are results from selected cytokines or chemokines at the TB relapse endpoint from study 2. Data are the means ± SEM with statistically significant differences compared to non-infected controls designated as follows: *p < 0.05. Significant differences between the indicated infection groups are shown as #*p* < 0.05; ##*p* < 0.01.

To identify the immune signature of HIS mice in the TB relapse phase, in the presence or absence of HIV co-infection, multiplex ELISA assessment of lung, liver, and plasma was performed on animals from study 2. In general, HIV mono-infection did not significantly affect pulmonary cytokines and chemokines production, compared to non-infected animals (Figure 6). Lung of mice with TB relapse was characterized by moderate increases in several cytokines and chemokines with important roles in host immunity to *Mtb* (e.g., IL-1β, TNF-α, IL-17, and IFN-γ) and promotion of HIV pathogenesis (IL-1β, TNF-α, IL-6, and CCL2). Additional chemokines with roles in host defense against *Mtb* and HIV (CXCL10 and CCL4) were also elevated in lung, although increases in CCL4 did not reach significance. Expression of CCL2 remained elevated in lung of animals with co-infection while moderate, though non-significant, reductions of IL-1β and IL-6 were observed compared to the TB group. Activation of IL-17 in mice with TB relapse, however, was significantly suppressed due to HIV infection.

Several cytokines and chemokines were at or below the limit of detection in the lung including IL-2, IL-4, IL-5, IL-7, and IL-15. Increased G-CSF, and a trend (*p* = 0.06) for increased IL-10, was observed in lung of mice with HIV mono-infection as shown in Supplemental Figure 3. The levels of other detectable analytes did not significantly differ among treatment groups.

### Cytokine and Chemokine Signatures in Liver and Periphery

The cytokine/chemokine profile of the liver tissue was also assessed to identify microenvironment signatures associated with hepatic TB relapse or *Mtb*-driven viral replication. In general, the liver microenvironment was less activated compared to the lung as shown in the treatment group comparisons in Supplemental Figure 4. Similar to the lung, the expression of several analytes (IL-2, IL-4, IL-5, IL-7, and IL-15) was below or near the limit of detection. A signal for several other individual cytokines was detectable and demonstrated that there were no significant differences among treatment groups for most analytes measured. Exceptions included GM-CSF, levels of which decreased in all infection groups while co-infection was associated with a significantly more marked suppression (Supplemental Figure 4). An interesting pattern of activation and suppression was observed for IL-1ra, CCL4, CXCL10, and IL-17 (Supplemental Figure 4). Increased production was observed in liver of mice from the TB group. Co-infection with HIV, however, was associated with a suppression of these cytokines/chemokines in comparison to TB (Supplemental Figure 4).

Plasma was further assessed to explore biomarkers that may reflect the disease and immune responses of the tissue compartments. As compared to the lung and liver, fewer cytokines and chemokines were above the limit of detection and the levels of detectable analytes were generally low (Supplemental Figure 5). Few cytokines were significantly altered due to treatment with the exception of activation of CXCL10 in the TB group. Some cytokines were observed to be decreased in all infection groups including IL-9, FGF, and PDGF. As observed in the lung and liver, CXCL10 was significantly reduced in the TB/HIV, compared to the TB, treatment group. TB/HIV was associated with a significant suppression of CCL4, G-CSF, and a trend toward suppression of TNF-α (*p* = 0.06), compared to TB.

## Discussion

TB recurrence due to relapse, reinfection, or reactivation of a latent *Mtb* infection continues to be a critical obstacle in the control and eradication of tuberculosis. HIV infection is an important contributing factor for: the current incidence of TB treatment failure or post-therapy relapse (Pulido et al., 1997; Crampin et al., 2010; Narayanan et al., 2010; Unis et al., 2014; Gadoev et al., 2017); the probability of death as a result of recurrent TB (Alvaro-Meca et al., 2014); and the development of *Mtb* drug resistance following relapse (Yoshiyama et al., 2004; McIvor et al., 2017). The challenges to investigations in human subjects and lack of experimental models has resulted in a poor understanding of the mechanisms for relapse TB in those with HIV.

Our work contributes, to the best of our knowledge, the first candidate small animal model of post-drug treatment relapse of TB amenable to the study of HIV co-infection. Use of HIS mice overcomes the challenge of human host restriction by HIV that otherwise limits investigations of TB relapse to correlative clinical studies of co-infected human subjects and extrapolations from studies of LTBI reactivation in SIV-infected NHP. Importantly, the HIS mouse model would also permit investigations of the CD4^+^T cell-independent mechanisms of disease due to HIV infection that are not reproduced through generalized immune suppression with aminoguanidine or glucocorticoids, *in vivo* CD4^+^T cell subset depletion, or use of mouse strains with immune defects (Scanga et al., 1999; Botha and Ryffel, 2002; Cheigh et al., 2010).

In the current study, HIV infection was implemented at the end of TB drug therapy to focus the investigation on the effects of HIV during the post-drug phase. This allowed us to reduce potentially confounding effects of chronic HIV on immunity and tissue mycobacterial burden at the end of TB chemotherapy on interpretation of results. HIV infections are an important risk for several TB disease scenarios in endemic regions (Tornheim and Dooley, 2018) including during development of active disease as well as during TB treatment and follow up. Acute HIV develops rapidly in the first few weeks after infection (Cohen et al., 2011) while chronic infection, including in those virally suppressed, perturbs the immune system (see reviews; Morou et al., 2014; Younas et al., 2016). Several other important clinical scenarios that could be modeled in HIS mice include: the effects of chronic HIV infection on the length of drug treatment needed to achieve clearance; the persistence of immune defects despite ART suppression of viral load; and the effects of ART and TB drug interactions on treatment windows and rates of relapse.

Our results demonstrate that paucibacillary TB can be generated in HIS mice following treatment with standard TB drugs and treatment timelines similar to the Cornell or Cornell-like approaches used in other murine models (McCune et al., 1966; Radaeva et al., 2005). The relapse kinetics we observed following HIV co-infection or high dose *Mtb* infection were more similar to those observed in genetically susceptible (e.g., I/St) as compared to resistant (e.g., C57BL/6) mouse strains (Radaeva et al., 2005). Our results suggest the infectious dose or human host genetics may result in different relapse outcomes, or different kinetics of relapse, regardless of the apparent development of paucibacillary disease observed following drug treatment. Based on our observations, 4 weeks of infection is sufficient to establish a bacterial burden that is paucibacillary following 8 weeks of drug treatment. The disease outcomes affected by HIV in the model may also be more discernible after lower dose infections (e.g., ≤10^2^ CFU). Further model development will be needed to determine the range of relapse kinetics in HIS mice developed from diverse human stem cell donors and in cord blood models. Once fully developed, these model systems would allow for hypothesis-driven investigations of disease susceptibility through generation of mice with stem cells from sources with known genetic backgrounds.

Post-drug TB relapse, or reactivation from latency, is often postulated to be the result of HIV-related or other immune disturbances which cause breakdown of an existing granuloma and permit dissemination (Diedrich and Flynn, 2011). These paradigms are generally well-supported by observations in NHP models of TB latency and latent TB reactivation due to immune suppression such as following TNF blockade therapy or SIV infection (Diedrich et al., 2010; Mehra et al., 2011; Foreman et al., 2016). In NHPs that have developed LTBI, necropsy reveals well-organized lesions persisting in the lung despite the absence of detectable bacilli (Hudock et al., 2017). In an elegant study of latent TB reactivation in the NHP due to TNF-α blockade, fluorescence labeling of metabolic hotspots along with PET CT imaging of granulomas demonstrated activity in previously affected areas of the lung (Lin et al., 2016). In that study, new granulomas did also develop simultaneously with, or in advance of apparent reactivation of the residual granulomatous areas, suggesting events beyond a breach of lesion containment also occur. To date, similar studies of post-drug relapse due to SIV infection have not been described in an NHP model.

Analysis of granulomatous inflammation outcomes related to *Mtb* containment following TB drug treatment is challenging in many murine models due to the limited development of granulomatous pathology. Compared to standard murine TB models, a strength of the HIS mouse is the spectrum of granulomas that develop including large necrotizing, and caseous lesions (Calderon et al., 2013; Nusbaum et al., 2016) with similarities to those observed in C3HeB/FeJ mice (Pan et al., 2005; Kramnik and Beamer, 2016). Lesions persist following TB drug treatment in C3HeB/FeJ, and other susceptible models such as the I/St, mouse (Kondratieva et al., 2018; Xu et al., 2019). In the current study, an apparent resolution of these granulomatous lesions was observed following TB chemotherapy in HIS mice. At relapse, the most consistent observation was that of moderate to significant interstitial inflammation and presence of AFB in these inflamed interstitial spaces. Our observations of pulmonary pathology in the HIS mouse thus further support the concept that TB relapse may also occur via mechanisms independent of granuloma integrity breakdown similar to those observed during LTBI reactivation in NHP (Lin et al., 2016). Further investigation is needed to determine if the outcomes observed in the HIS mouse are unique to the model or may represent a spectrum of events that occur in TB relapse in human lung and advanced animal models such as NHP. Our initial findings in the setting of co-infection suggest that HIV infection promotes greater mycobacterial proliferation in the post-chemotherapy stage independent of effects on granuloma integrity.

Pulmonary viremia was previously demonstrated to be increased in PLWH that had active TB compared to those without TB (Nakata et al., 1997). Our results are consistent with these observations and demonstrate the first preliminary evidence, to our knowledge, that these polymicrobial outcomes in the lung could be modeled in the HIS mouse. The observations that HIV+ cells localized to the sites of interstitial inflammation and *Mtb* proliferation further suggest potential mechanisms of microbial synergy in the lung. The inflammatory immune response that restrains *Mtb* propagation in lesions may recruit new targets and establish activation conditions favorable for HIV replication. Likewise, the suppression of immunity due to local effects of HIV infection could promote *Mtb* proliferation. Importantly, our results suggest that these tissue events of co-infection may differ from those in the periphery, as described in one report of human subjects (Nakata et al., 1997). We observed increased viral replication in the lung and liver in the setting of *Mtb* co-infection while plasma viral loads were similar among HIV and HIV/TB groups. These differences in viremia between the blood and tissues could suggest that blood-based diagnostics may not fully reflect the *in vivo* disease process in those with co-infection with regard to the impact on viral load or viral reservoirs.

Multiplex analysis of lung analytes from HIS mice with relapse demonstrated a moderate activation of many cytokines and chemokines including pro-inflammatory molecules. HIV infection is known to promote a pro-inflammatory bias in the infected host (Nou et al., 2016) while several inflammatory mechanisms are associated with increased *Mtb* pathogenesis (Tobin, 2015; Wallis and Hafner, 2015). In the current study, HIV mono-infection generally presented with only moderate and non-significant activation of cytokines and chemokines in the tissues and plasma compared to non-infected animals. Infection with *Mtb* in the post-drug relapse phase, however, was associated with pulmonary activation of several pro-inflammatory cytokines and chemokines that have established roles in enhancing HIV replication including IL-1β, IL-6, TNF-α, and CCL-2 (Swingler et al., 1994; Kumar et al., 2013; Nou et al., 2016; Pasquereau et al., 2017). CCL2 was recently shown to further support HIV pathogenesis by promoting virion release from infected host cells (Ajasin et al., 2019). Increased proliferation of HIV in lung of persons with *Mtb* co-infection has been previously associated with increased TNF-α and CCL2 levels in bronchoalveolar lavage (Mayanja-Kizza et al., 2009; Kumar et al., 2013). Further exploration in HIS models may demonstrate if the production of these pro-viral molecules at sites of *Mtb*-driven inflammation favor HIV replication through a variety of potential mechanisms including recruitment of inflammatory target cells, direct activation of the HIV LTR, and augmented virion release from productively infected host cells.

Effector cytokines with anti-mycobacterial function (e.g., IFN-γ, TNF-α, and IL-17) (Ernst, 2012) were also observed to be moderately activated in the lung microenvironment at relapse. Co-infection with HIV did not significantly reduce the activation of IFN-γ or TNF-α at *Mtb* regrowth endpoints, suggesting lack of a generalized immune suppression. In contrast, HIV infection reduced production of pulmonary IL-17 following activation by *Mtb* regrowth. These preliminary results may suggest an interesting candidate mechanism of microbial synergy for further exploration. The Th17 subset of CD4^+^T cells are very permissive to HIV infection due to lack of RNases (Christensen-Quick et al., 2016). Impairment and depletion of Th17 cells in PLWH is also known to occur in the early stages of infection (El Hed et al., 2010; Prendergast et al., 2010; Murray et al., 2018). IL-17 produced by multiple cell populations is important for host immunity to *Mtb* (Lockhart et al., 2006; Umemura et al., 2007; Okamoto Yoshida et al., 2010; Torrado and Cooper, 2010; Shen and Chen, 2018) and is elevated in persons with latent TB compared to healthy controls (Coulter et al., 2017; Devalraju et al., 2018). In support of our observations, reduced IL-17 was also associated with TB regrowth in a BALB/c mouse model of post-drug TB relapse (de Steenwinkel et al., 2013).

The overall pattern of cytokine and chemokine activation in the liver was similar, but muted, compared to the lung. This outcome is consistent with the lower pathogen burden in this site of dissemination. Similarly, even fewer significant differences in cytokine and chemokine levels were observed in the plasma at relapse. An interesting exception was the activation of CXCL10 observed in the lung, liver, and plasma of animals in the TB relapse group. CXCL10 has been previously described as a biomarker capable of distinguishing active and latent TB and predicting the risk for TB chemotherapy failure (Hong et al., 2014; Wergeland et al., 2015). In contrast to the observations in human subjects that CXCL10 predicted TB disease state irrespective of HIV status (Wergeland et al., 2015), we observed a significant reduction of CXCL10 in the liver and plasma, but not the lung, of HIS mice in the setting of co-infection. Further studies are needed to validate the biomarker potential of CXCL10 and other immune molecules to inform TB relapse outcomes and identify the effect of HIV on these biomarkers. Our preliminary findings support the potential for this small animal model to reproduce important immune outcomes in a system where mechanistic investigations of the lung and other tissue compartments can be undertaken and therapeutic interventions can be tested.

There are several challenges to use of HIS mice that should be noted despite the obvious advantages for study of HIV and co-infections such as HIV and *Mtb*. Animals are expensive to generate and maintain, and often require 2 to 3 months for development of immune reconstitution following irradiation and engraftment of human stem cells. The chimeric nature of the system means that immune outcomes include both human adaptive and innate, as well as murine innate, responses. There are additionally suboptimal development of some immune responses such as humoral immunity in HIS mice (Karpel et al., 2015). In the NSG mouse strain used to generate BLT mice in the current study, development of GVHD is a known issue (Greenblatt et al., 2012) that led to losses in animal numbers in the prolonged study design required to reproduce TB treatment and relapse as reported here. Recent regulatory and legal issues have further restricted production of, and funding for, the BLT HIS model in many states and institutions. Nonetheless, the outcomes of the current study are important to guide development of models of co-infection including HIV-associated TB relapse in newer HIS models. Cord blood stem cells can generate HIS mice with similar reconstitution efficiency to the BLT (Wunderlich et al., 2018) while further refinements in these models can expand different immune compartments and reduce development of GVHD (Iwabuchi et al., 2018).

In conclusion, our findings describe a candidate small animal model of TB relapse in the setting of HIV infection that reproduces aspects of clinical disease, including microbial synergy and immune activation, observed in co-infected persons. Long term, HIS mouse models of TB relapse may serve an important pre-clinical role for discovery and translation of therapeutics prior to final evaluation in NHP models. Investigations in models capable of supporting co-infections are critical to improving TB treatment outcomes in HIV+ persons.

## Data Availability Statement

All datasets generated for this study are included in the article/Supplementary Material.

## Ethics Statement

The animal study was reviewed and approved by the University of Texas Medical Branch Institutional Animal Care and Use Committee.

## Author Contributions

Investigations into the feasibility and application of the HIS mouse to study TB relapse in the setting of HIV were carried out by MH, TS, RN, KN, SC, JR, ME, JL, BG, and JE. Data generated from these investigations was analyzed by MH, RN, KN, SC, RH, JA, BG, and JE. MH and JE prepared the manuscript and upon careful review by TS, RN, KN, SC, JR, and BG. ME edited the document for submission.

### Conflict of Interest

The authors declare that the research was conducted in the absence of any commercial or financial relationships that could be construed as a potential conflict of interest.
