# Supplementary material for: Small Animal Model of Post-chemotherapy Tuberculosis Relapse in the Setting of HIV Co-infection
Source: Front Cell Infect Microbiol. 2020 Apr 16;10:150. doi: 10.3389/fcimb.2020.00150 (PMC7176873; doi:10.3389/fcimb.2020.00150)
Supplement: Supplementary file 1 [file Data_Sheet_1.PDF]

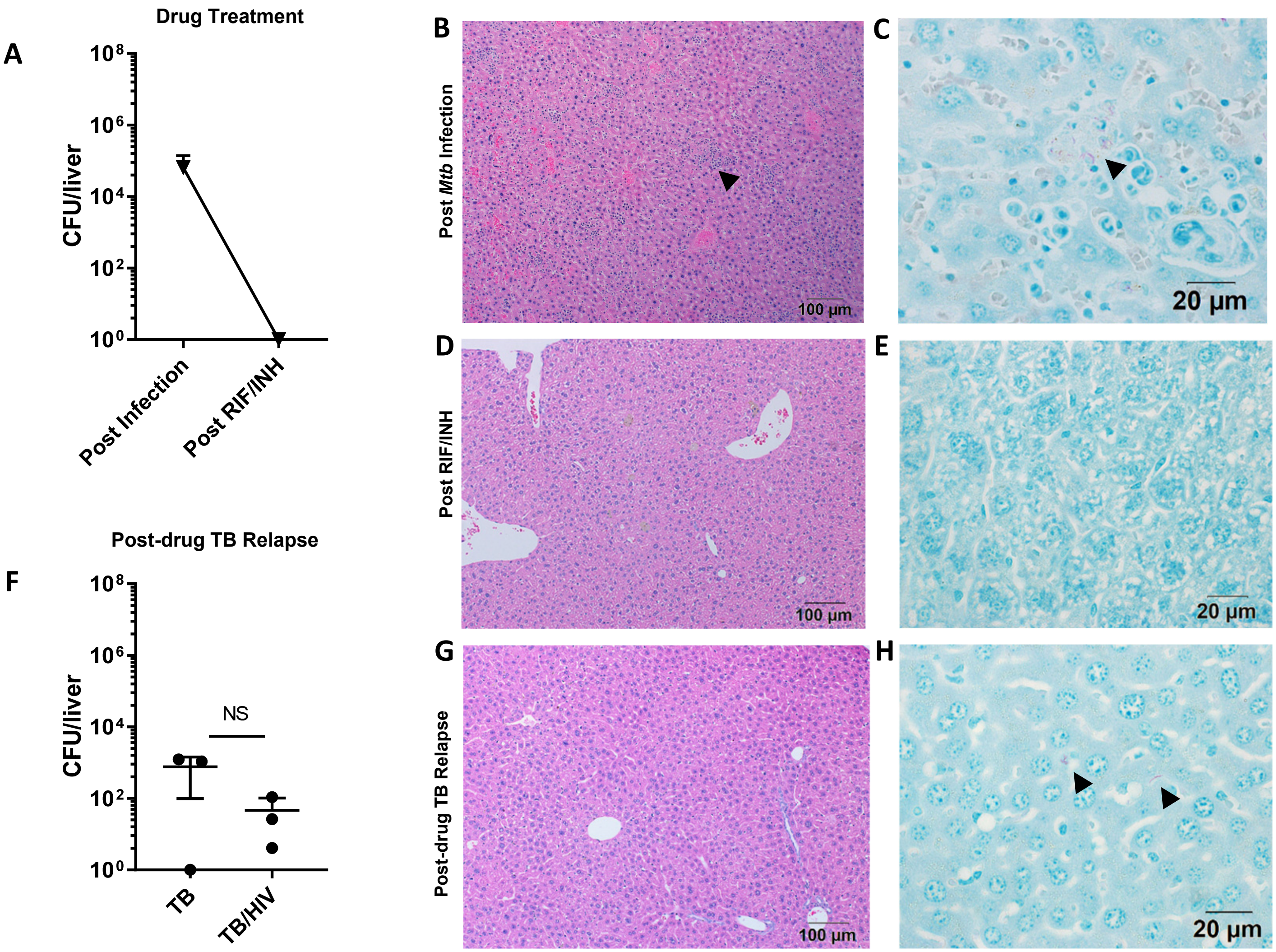

Supplemental Figure 1. TB relapse in the liver following low dose *Mtb* infection. Infection, drug treatment and TB relapse outcomes in liver of HIS mice in study 1. **(A)** CFU in the liver 8 weeks after i.n. infection with *Mtb* and after 8 weeks of TB chemotherapy demonstrating a paucibacillary TB state. **(B)** Small inflammatory foci containing **(C)** AFB in the liver of mice following infection with *Mtb*. **(D)** Mostly non-remarkable liver tissue observed following TB chemotherapy which **(E)** lack detectable AFB. **(F)** Hepatic mycobacterial burden (CFU) at the relapse endpoint 8 weeks after mock or HIV infection in the mock (TB) or HIV (TB/HIV) co-infection groups. **(G)** Similar and mostly non-remarkable liver observed in mice from both TB and TB/HIV groups in the relapse phase in which **(H)** rare AFB are observed.

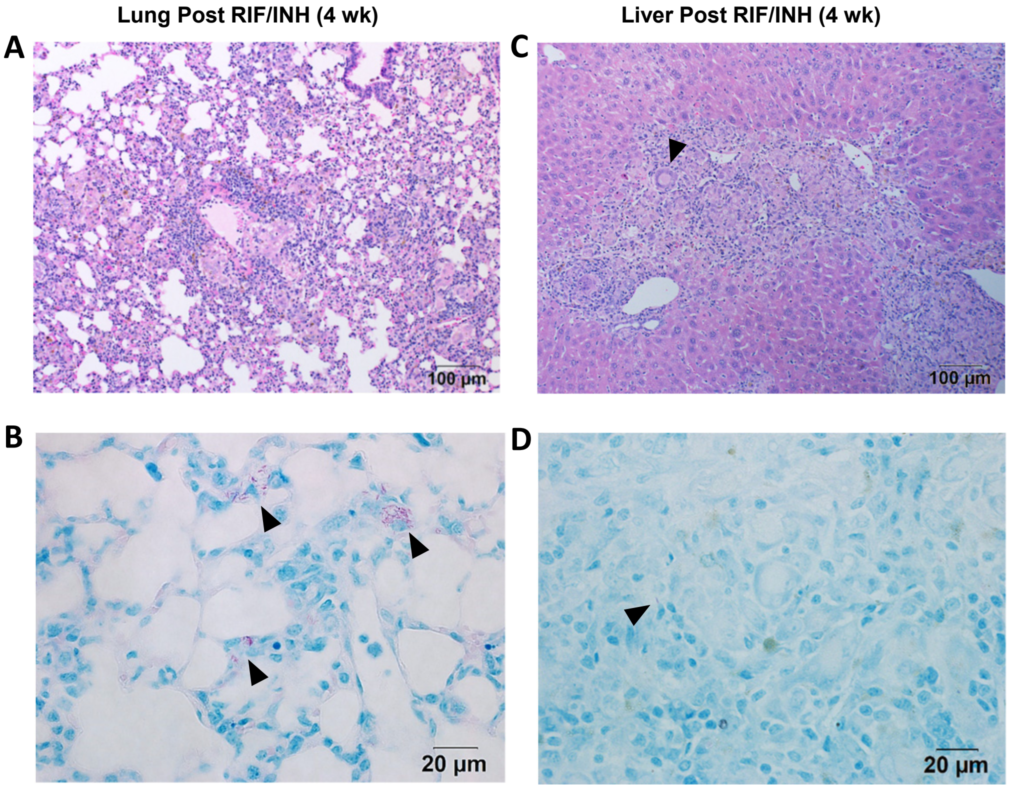

Supplemental Figure 2. Pulmonary histology during drug treatment. Histological appearance of tissue from an infected and drug treated mouse at 4 weeks post RIF and INH treatment in study 2. Mice were infected with 10^3 *Mtb* bacilli i.n. and infection progressed for 4 weeks followed by 8 weeks of chemotherapy with RIF and INH. **(A)** Histological appearance of lung at 4 weeks post RIF and INH treatment demonstrating diffuse areas of inflammation and **(B)** pockets of AFB (arrowhead). **(C)** Histological appearance of the liver at 4 weeks post RIF and INH treatment demonstrating lesions with multi-nucleated giant cells (arrowheads) and **(D)** rare AFB (arrowhead).

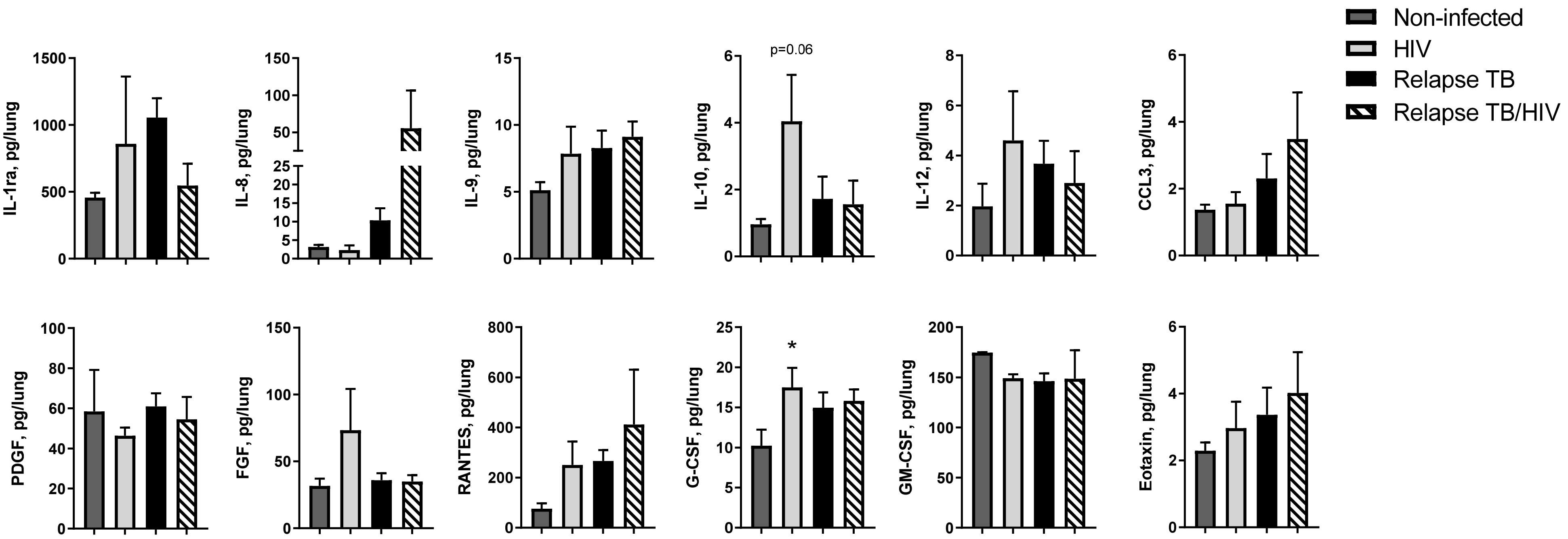

Supplemental Figure 3. Expanded analysis of pulmonary inflammation at TB relapse. Supernatants were harvested from disrupted lung tissue to assess differences in the immune microenvironment at TB relapse, in the presence or absence of HIV. Analysis of lung cytokines and chemokines was performed with a human multiplex ELISA (Bio-rad Bio-plex Pro^{TM} human cytokine 27-plex kit). Shown are the additional lung cytokine and chemokine outcomes from study 2 that correspond with those displayed in Figure 6. Data are the means $\pm$ SEM with statistically significant differences compared to non-infected controls designated by *, $p<0.05$.

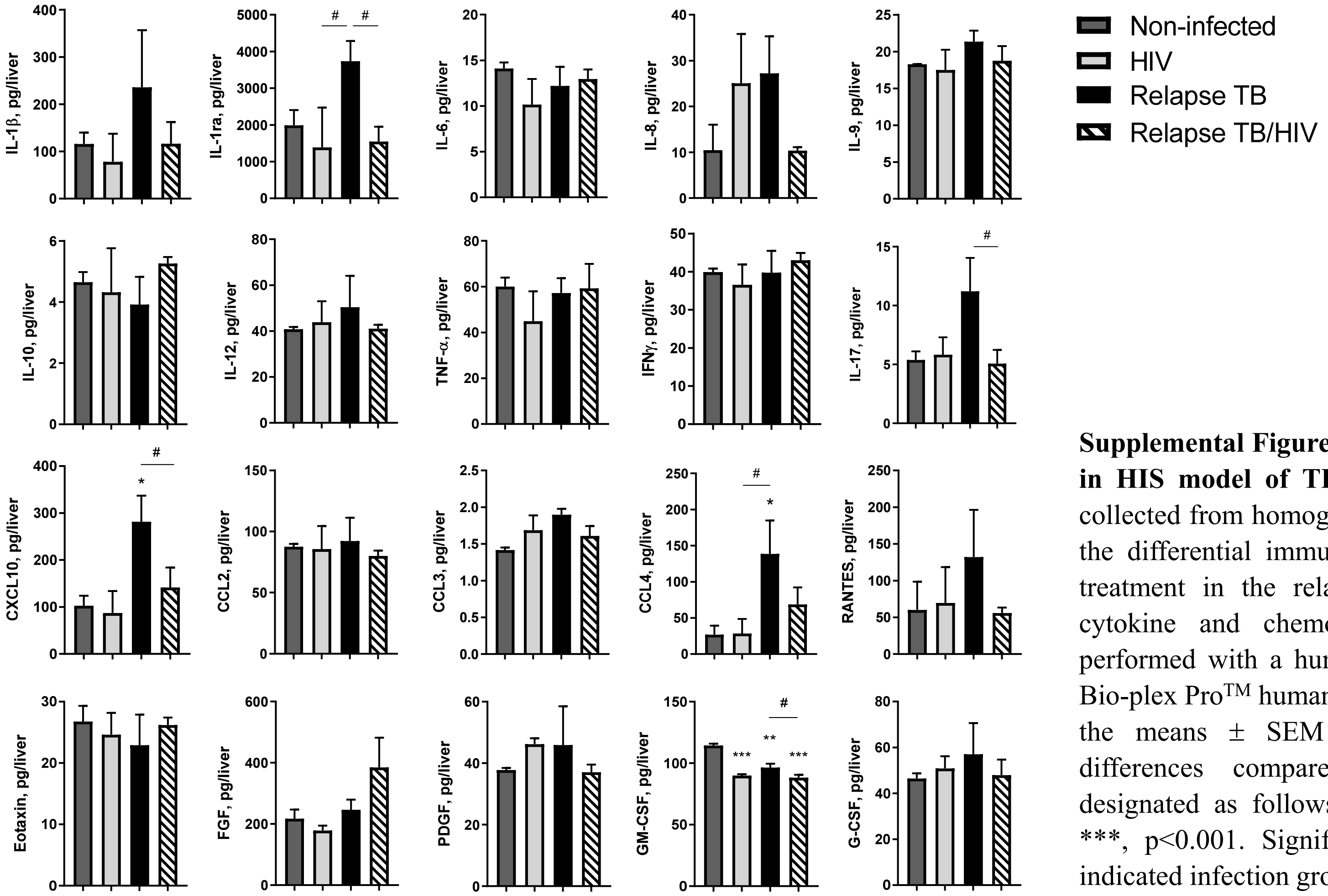

Supplemental Figure 4. Hepatic immune response in HIS model of TB relapse. Supernatants were collected from homogenized liver tissue to determine the differential immune responses observed due to treatment in the relapse phase. Analysis of liver cytokine and chemokine expression levels was performed with a human multiplex ELISA (Bio-rad Bio-plex Pro^{TM} human cytokine 27-plex kit). Data are the means ± SEM with statistically significant differences compared to non-infected controls designated as follows: *, p<0.05, **, p<0.01; and ***, p<0.001. Significant differences between the indicated infection groups are shown as #, p<0.05.

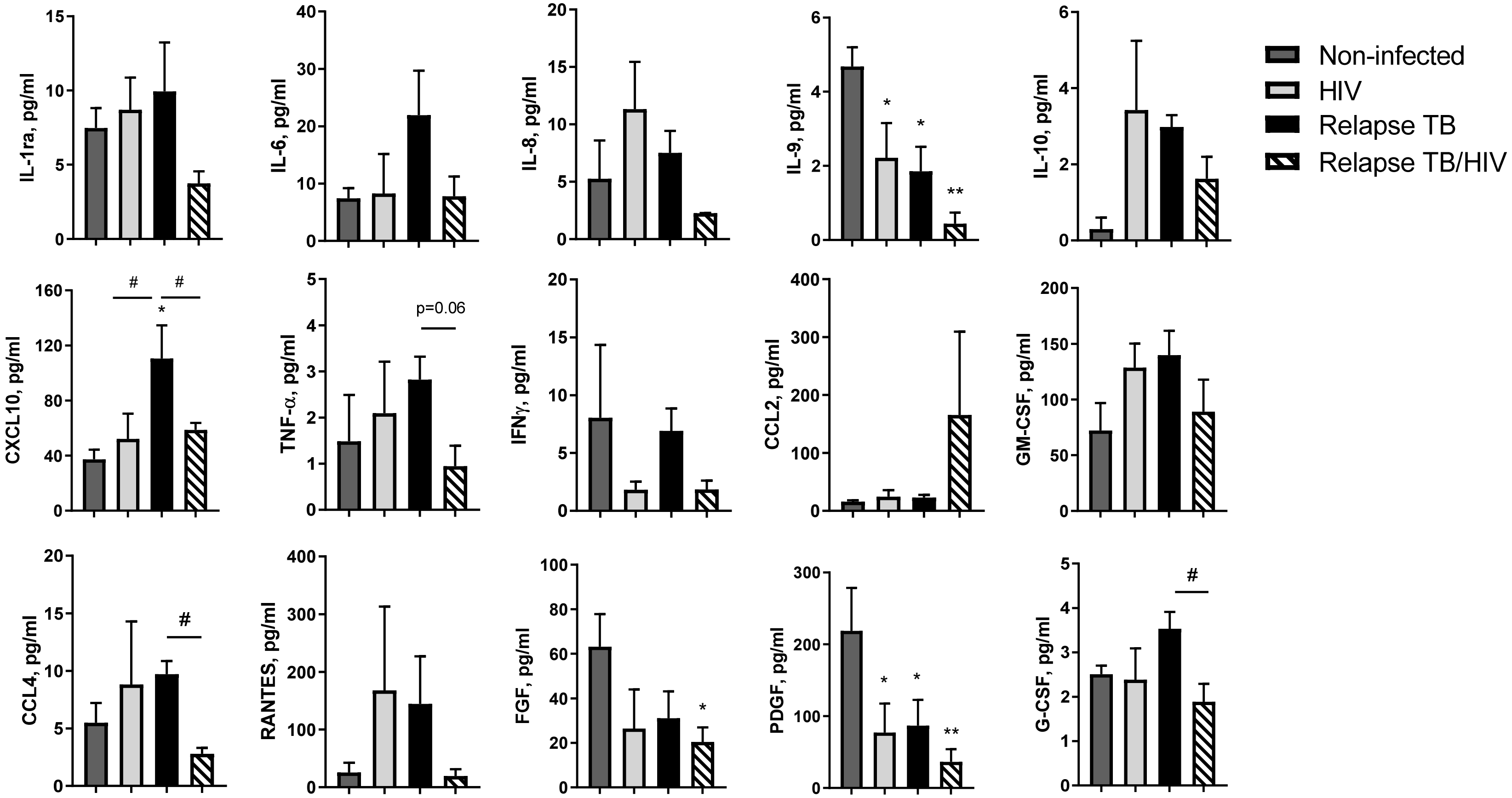

Supplemental Figure 5. Systemic cytokine and chemokine profile of HIS mice in the TB relapse phase. Plasma was separated from the blood and analyzed by human multiplex ELISA (Bio-rad Bio-plex Pro™ human cytokine 27-plex kit) to identify potential biomarkers that may reflect tissue compartment disease. Changes in measurable cytokines and chemokines as a result of treatment are shown with significant differences compared to non-infected controls designated as: *, p<0.05 and **, p<0.01. Significant differences between the indicated infection groups are shown as #, p<0.05.
